# Genetic liability to rheumatoid arthritis on autism and autistic traits: polygenic risk score and Mendelian randomization analyses

**DOI:** 10.1038/s41398-021-01772-2

**Published:** 2022-01-12

**Authors:** Amanda Ly, Beate Leppert, Dheeraj Rai, Hannah Jones, Christina Dardani, Evie Stergiakouli

**Affiliations:** 1 Centre for Academic Mental Health, Bristol Medical School, University of Bristol, Bristol, UK; 2 MRC Integrative Epidemiology Unit, Bristol Medical School, University of Bristol, Bristol, UK; 3 Population Health Sciences, Bristol Medical School, University of Bristol, Bristol, UK; 4 National Institute for Health Research Bristol Biomedical Research Centre, University Hospitals Bristol NHS Foundation Trust, University of Bristol, Bristol, UK; 5 Avon and Wiltshire Partnership NHS Mental Health Trust, Bristol, UK

**Keywords:** Autism spectrum disorders, Predictive markers

## Abstract

Higher prevalence of autism in offspring born to mothers with rheumatoid arthritis has been reported in observational studies. We investigated (a) the associations between maternal and offspring’s own genetic liability for rheumatoid arthritis and autism-related outcomes in the offspring using polygenic risk scores (PRS) and (b) whether the effects were causal using Mendelian randomization (MR). Using the latest genome-wide association (GWAS) summary data on rheumatoid arthritis and individual-level data from the Avon Longitudinal Study of Parents and Children, United Kingdom, we constructed PRSs for maternal and offspring genetic liability for rheumatoid arthritis (single-nucleotide polymorphism [SNP] *p*-value threshold 0.05). We investigated associations with autism, and autistic traits: social and communication difficulties, coherence, repetitive behaviours and sociability. We used modified Poisson regression with robust standard errors. In two-sample MR analyses, we used 40 genome-wide significant SNPs for rheumatoid arthritis and investigated the causal effects on risk for autism, in 18,381 cases and 27,969 controls of the Psychiatric Genetics Consortium and iPSYCH. Sample size ranged from 4992 to 7849 in PRS analyses. We found little evidence of associations between rheumatoid arthritis PRSs and autism-related phenotypes in the offspring (maternal PRS on autism: RR 0.89, 95%CI 0.73–1.07, *p* = 0.21; offspring’s own PRS on autism: RR 1.11, 95%CI 0.88–1.39, *p* = 0.39). MR results provided little evidence for a causal effect (IVW OR 1.01, 95%CI 0.98–1.04, *p* = 0.56). There was little evidence for associations between genetic liability for rheumatoid arthritis on autism-related outcomes in offspring. Lifetime risk for rheumatoid arthritis has no causal effects on autism.

## Introduction

Autism spectrum disorders, henceforth autism, are neurodevelopmental conditions characterised by difficulties with social reciprocity, and by restricted, repetitive patterns of behaviour and interests [1]. The worldwide prevalence of autism is estimated to be 1–2% according to large-scale surveys [1], but there are also individuals affected by autistic traits who are undiagnosed. Although the aetiology of autism is not well understood, there is consensus that both genetic and environmental exposures, and their interplay are involved in autism development [2]. Twin and population-based studies have estimated autism heritability to be between 64 and 91% [3]. The contribution of genetics to autism aetiology is complex; large studies have shown that rare genetic variants with large effect sizes as well as common genetic variants with small effect sizes confer risk to autism [4]. The cumulative effect of these common genetic variants is an important risk factor for development of autism [4].

Rheumatoid arthritis is a highly heritable, common autoimmune disorder that is chronic and systemic in nature [5]. It affects between 0.5 and 1% of the general population of Northern European/North American heritage, with females at higher risk. Some observational studies have explored the potential association between maternal autoimmune diseases and autism in offspring, including rheumatoid arthritis [6, 7]. Maternal autoimmune diseases as a composite exposure are associated with increased risk of autism in offspring [6, 8]. As one of the more common autoimmune diseases, we focussed on the evidence base assessing maternal rheumatoid arthritis and autism; previous studies have found evidence of the associations between rheumatoid arthritis and autism in the offspring [6, 7, 9–11]. One of these studies, a nationwide, Danish cohort study, found strong evidence for an association of maternal rheumatoid arthritis with an increased risk of autism in offspring, with weaker evidence for an association between paternal rheumatoid arthritis and autism in offspring [9].

Two possible mechanisms have been proposed for maternal rheumatoid arthritis to exert an effect on risk of autism in offspring. The first one is through pregnant women having elevated levels of maternal autoantibodies and cytokines, which could increase inflammatory activity in the intrauterine environment [7, 12]. Challenging *in utero* conditions due to increased maternal inflammation could alter healthy foetal brain development, plausibly leading to autism [13, 14]. This hypothesis has also been articulated in the context of other maternal autoimmune disorders, but without robust findings [6, 8]. An alternative hypothesis is that offspring inherit genetic liability to rheumatoid arthritis, which could result in greater susceptibility to immune dysregulation and increased neuroinflammation in the brain [9]. Immune irregularities in autism have been identified [15], and family history of autoimmune disease, including rheumatoid arthritis, is associated with increased risk of autism [16]. However, whether such activity is implicated in pathogenesis is not clear. Large, prospective cohort studies are needed to strengthen the evidence base.

Genome-wide association studies (GWASs) have discovered numerous single-nucleotide polymorphisms (SNPs), or common genetic variants, with small effect sizes that are associated with autism and rheumatoid arthritis at genome-wide significance level [17, 18]. Despite some observational evidence suggesting that rheumatoid arthritis is associated with increased risk of autism, the two traits have found to have a negative genetic correlation (−0.134 [SE 0.06], p value 1.72 × 10^−2^) [18]. The possible shared genetic liability between the disorders reflects a need for further investigation into the genetic relationship between them. Polygenic risk scores (PRSs), derived from summing an individual’s trait-associated SNPs, can be used to investigate the downstream outcomes of an individual’s genetic liability to a trait of interest [19]. Although use of PRS can provide causal evidence, one shortcoming is the absence of a formal test to assess pleiotropy. Further triangulating investigations with Mendelian randomization (MR) can be used to infer causality; such a method utilizes genetic variants identified through GWAS as proxies for modifiable exposures. Since genetic variants are randomly assigned at conception, using MR mitigates the effects of unmeasured confounding by environmental exposures and reverse causation [20]. Pleiotropy can be tested.

To our knowledge, the association between genetic liability to rheumatoid arthritis and autism has not been investigated in a large, population-based cohort. In this study, we aimed to assess (a) the associations between maternal genetic liability for rheumatoid arthritis and risk of autism and autistic traits in the offspring; (b) the offspring’s own genetic liability for rheumatoid arthritis and risk of autism and autistic traits (in the absence of paternal genetic data), and in a separate independent study, (c) assess whether the effect is causal using MR.

## Methods

Participants were mothers and index children from the Avon Longitudinal Study of Parents and Children (ALSPAC) birth cohort. The ALSPAC study has been described elsewhere [21, 22]. Briefly, pregnant women with expected delivery dates between 1 April 1991 and 31 December 1992 from the former County of Avon, United Kingdom, were recruited. Initially, 14,541 pregnancies were enroled, with 14,062 subsequent births and 13,988 children who were alive at 1 year of age. There was a booster recruitment period when the oldest-child participants were approximately 7 years of age, which enabled eligible cases to join if they had not done so originally. The total sample size for analyses using any data collected after the age of seven is 15,454 pregnancies, resulting in 15,589 foetuses. There were 14,901 infants alive at 1 year of age. Since recruitment, a range of data have been collected via self-administered questionnaires, biological samples, clinical assessments, and linkage to medical and educational records, among others, during multiple data-collection phases. Further information on the collection of genetic data is provided in the Supplementary materials. Please note that the study website contains details of all the data that are available through a fully searchable data dictionary and variable search tool (http://www.bristol.ac.uk/alspac/researchers/our-data/). Ethical approval for the study was obtained from the ALSPAC Ethics and Law Committee and the Local Research Ethics Committees (http://www.bristol.ac.uk/alspac/researchers/research-ethics/). Consent for biological samples has been collected in accordance with the Human Tissue Act (2004). Informed consent for the use of data collected via questionnaires and clinics was obtained from participants following the recommendations of the ALSPAC Ethics and Law Committee at the time.

We identified autism in ALSPAC child participants using a multisource approach [23]. This included: review of children with a statement for special educational provision and clinical record review for children flagged as having a developmental disorder—these cases were validated by a consultant paediatrician using the International Statistical Classification of Disease, 10th revision [23]. Other sources: mother’s response to the question “Have you ever been told that your child has autism, Asperger’s syndrome or autism?” when their child was 9 years old; response to questions on diagnosed conditions in questionnaires administered when the child was between 6 months and 11 years old in unstructured text; via ad hoc parental reports of an autism or Asperger syndrome diagnosis, and identification via the educational system as requiring learning support due to autism by age 16. Autism cases were also cross-validated against autistic trait measures [24, 25], and were previously found to be associated with PRSs of autism.

ALSPAC investigators have collected 93 measures related to autistic features up until 11 years old from child participants. Of these, 88 were derived from self-completed questionnaires, with the remainder from carer-completed questionnaires. The four autistic measures, described as traits in this paper, were the strongest predictors of autism [26]. These traits were: social and communication difficulties as measured by the Social Communications Disorder Checklist (SCDC, assessed at 91 months); coherence from the Children’s Communication Checklist (assessed at 115 months), repetitive behaviours on the Repetitive Behaviour scale (assessed at 69 months); and sociability as part of the Emotionality, Activity and Sociability Temperament scale (assessed at 38 months). We generated binary variables for these traits with cutoffs selected to represent the top ‘high risk’ decile of each trait distribution, coded 1 for ‘high risk’ and 0 for ‘lower risk’.

### Polygenic risk score calculation

To capture maternal and offspring’s own genetic liability for rheumatoid arthritis, standardized PRSs were constructed for each ALSPAC participant using the results of Okada et al.’s rheumatoid arthritis GWAS on individuals of European ancestry [17], and PRSice v1.25 [27]. The rheumatoid arthritis GWAS contained 14,361 cases of rheumatoid arthritis and 43,923 controls [17]. There were no overlapping participants between ALSPAC and the rheumatoid arthritis GWAS sample. SNPs from autosomal chromosomes were used to construct PRS at 12 GWAS *p*-value thresholds: 0.5, 0.4, 0.3, 0.2, 0.1, 0.05, 0.01, 1 × 10^−3^, 1 × 10^−4^, 1 × 10^−5^, 1 × 10^−6^, and 1 × 10^−7^, with 0.05 as the *p*-value threshold that was used in the main analysis. SNPs from the major histocompatibility complex (MHC) region (Chr6:26Mb–33Mb) were excluded [27]. Linkage disequilibrium (LD) clumping was performed using a *r*^2^ threshold of 0.1 within a sliding window of 1000 kb to ensure limited correlation between SNPs.

As a secondary analysis, we assessed whether genetic liability for juvenile idiopathic arthritis (JIA) is associated with autism/autistic traits through possible mechanisms as outlined in the 'Introduction', albeit from an earlier life stage. Although there is some research demonstrating that adult and juvenile arthritis as a composite variable is associated with autism in offspring, the study was likely underpowered to investigate JIA on its own [8]. With a greater available sample size in our study, JIA PRSs were created with the methods described above, using the results from a JIA GWAS comprising 2816 individuals with JIA and 13,056 controls of European ancestry [28].

### Instrumental variables for 2-sample Mendelian randomization analysis

Rheumatoid arthritis genetic instruments were identified using results of the rheumatoid arthritis GWAS that were also used for constructing PRSs [17]. For the MR analysis, rheumatoid arthritis genetic instruments were defined as SNPs that met a GWAS *p*-value threshold of 5 × 10^−8^. LD clumping was performed using a *r*^2^ threshold of 0.001 within a 10,000-kb sliding window, followed by harmonisation with the autism GWAS results to exclude SNPs that were palindromic or ambiguous due to lack of data on effect allele frequencies. Forty rheumatoid arthritis-associated SNPs were identified, and their effects on autism were calculated, producing effect sizes and SEs. There were no overlapping participants between the autism and rheumatoid arthritis GWAS samples, which we manually checked by assessing the list of consortia included in each GWAS. The autism GWAS results were from the Psychiatric Genomics Consortium (PGC) and the Lundbeck Foundation Initiative for Integrative Psychiatric Research (iPSYCH) GWAS, which comprised 18,382 cases and 27,969 controls of European ancestry [18]. We were not interested in bidirectionality as our research questions focus on autism and autistic traits as outcomes.

As a secondary analysis to assess the causal effects of genetic liability for JIA on autism, we identified SNPs using the same JIA GWAS results that were used for constructing PRSs [28]. The genetic instruments were identified following LD clumping of the JIA GWAS (*r*^2^ = 0.001, kb=10,000). This resulted in 6 SNPs that associated with JIA at *p* < 1 × 10^−7^.

### Statistical analysis

We estimated the frequency of autism diagnosis and autistic traits in those who were included in PRS analyses to establish how many index children were affected. Modified Poisson regression with robust standard errors was used to assess the associations between (i) maternal rheumatoid arthritis PRS; (ii) offspring rheumatoid arthritis PRS, and the 5-binary autism-related offspring phenotypes in the ALSPAC cohort. Analyses were adjusted for 10 genetic principal components (PCs) to account for population stratification. The associations between maternal JIA PRSs and autism-related offspring phenotypes were also tested. Stata v16 was used for statistical analysis [29]. Computer code requests can be directed to the corresponding author AL.

MR uses genetic variants as instrumental variables (IV) to proxy for modifiable exposures [20]. The assumptions of MR are (i) the IV is robustly associated with the exposure of interest, (ii) the IV is not associated with measured and unmeasured confounding factors, and (iii) the IV is associated with the outcome via the exposure only (exclusion restriction criteria). To test for possible causal relationships, unconfounded by environmental factors, we conducted two-sample MR analyses using genetic variants identified through the rheumatoid arthritis GWAS as described previously.

Three MR methods were used as they differ in their assumptions with respect to instrument validity: inverse-variance weighting (IVW), MR Egger regression, and weighted median. The IVW method assumes that all instruments are valid for the exposure of interest, i.e., that horizontal pleiotropy (whereby the instruments for the exposure of interest are associated with the outcome indirectly through traits other than the exposure) is not present or is balanced. The MR-Egger method relaxes this assumption, and formally tests for the presence of horizontal pleiotropy, however, this method requires more statistical power. The weighted-median method produces consistent effect estimates if at least 50% of the exposure-associated SNPs used as instruments are valid.

### Sensitivity analysis

To assess the differences between ALSPAC participants with and without genetic data, we compared the samples with respect to a range of sociodemographic characteristics. There was evidence for differences between the comparison groups with higher prevalence of autism cases and socioeconomic status (SES), and on average, lower prevalence of maternal postpartum depression and maternal smoking and higher maternal age in those who had genetic data. There were no differences in prevalence of autistic traits. Further details can be found in Table S1.

To explore the possibility of bias due to attrition, we also assessed the relationship between genetic liability to autism and participation in follow-up data collections. Previously having been studied in the ALSPAC cohort using data from an older, smaller autism GWAS, we used the same variables and methods as *Taylor et al*. [30]. That is, we constructed PRS using results of the most recent autism GWAS [18] for mothers and for their children at multiple *p*-value thresholds as our exposures. For mothers, the outcomes of interest were total participation, both in clinics from 2008 and total participation in questionnaires that the biological mother has answered about herself (maximum score 19). For the children, the outcomes were: total participation in questionnaires, both child-based and child-completed (max score 48), and total participation in questionnaires and clinics (max score 57). We used linear regression with robust standard errors to account for the non-normal distribution of the outcomes of interest. For the child-based analyses, we fit models adjusted with no covariates, and with 10 genetic PCs and sex. For the mother-based analyses, we fitted 2 models, one adjusting for 10 genetic PCs, and one without.

We also performed numerous sensitivity analyses to test assumptions in the MR analyses. These are further detailed in the Supplementary materials.

## Results

In genotyped child participants, there were 101 (1.29%) autism cases. In addition, there were 518 (9.5%) participants with the highest scores that indicate most difficulty with social communication, 554 (9.95%) with coherence, 385 (6.93%) with repetitive behaviours, and 716 (11.59%) with very reduced sociability temperament. The results from additional descriptive analyses are reported in Table S1.

Table 1 illustrates the results from measuring the effect of mother’s and offspring’s own genetic risk for rheumatoid arthritis (based on SNPs related to rheumatoid arthritis at *p* = 0.05) on autism-related phenotypes in offspring. We found little evidence of the association between genetic liability for rheumatoid arthritis and autism or autism traits.

**Table 1:** Associations between PRSs and autism-related phenotypes at GWAS *p*-value threshold 0.05, adjusted for 10 genetic principal components.

	Rheumatoid arthritis PRS for mothers			Rheumatoid arthritis PRS for offspring		
Outcome	*N*	RR (95% CIs)	*P*	*N*	RR (95% CIs)	*P*
*Autism cases*	7685	0.89 (0.73–1.07)	0.21	7849	1.11 (0.88–1.39)	0.39
*SCDC*	5071	0.98 (0.90–1.07)	0.95	5450	1.02 (0.94–1.10)	0.67
*Coherence*	4992	0.95 (0.87–1.03)	0.21	5568	1.01 (0.94–1.09)	0.76
*Repetitive behaviour*	5249	0.96 (0.86–1.06)	0.40	5557	1.06 (0.97–1.17)	0.21
*Sociability*	5965	0.96 (0.89–1.03)	0.27	6176	0.99 (0.92–1.06)	0.75

*PRSs* polygenic risk scores, *GWAS* genome-wide association study, *N* sample size in analysis, *RR* relative risk, (*95% CIs*) 95% confidence intervals, *P* *p*-value, *SCDC* social communication difficulties as measured by the Social Communications Disorder Checklist.

In further analyses using PRSs for rheumatoid arthritis constructed at different *p*-value thresholds, we found no strong evidence of the associations between maternal or child genetic liability for rheumatoid arthritis and autism-related phenotypes. For further details on effect sizes and 95% CIs, please refer to Tables S2–S6.

**Fig. 1:**
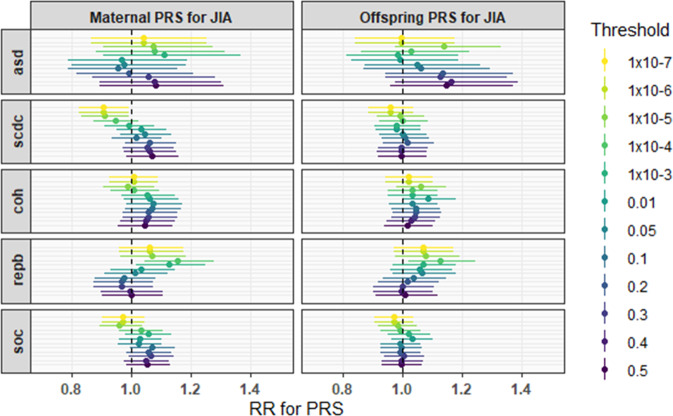
Associations between PRSs for juvenile idiopathic arthritis and autism-related phenotypes, adjusted for ten genetic principal components, at all SNP p-value thresholds. PRS polygenic risk scores, JIA juvenile idiopathic arthritis, RR relative risk, asd autism cases, SCDC social communication difficulties, coh coherence issues, repb repetitive behaviour, soc sociability temperament, Threshold SNP p-value threshold for PRS construction.

The results from assessing the associations between mother’s and offspring’s genetic liability for JIA and autism-related phenotypes at different *p*-value thresholds are illustrated in Fig. 1. We found little evidence of the associations at the main-analysis *p*-value threshold of 0.05. However, there was evidence for a protective effect of mother’s PRS and social communication difficulties at the most stringent GWAS *p*-value thresholds (RR 0.91 [95% CI 0.83, 0.99], *p* = 0.03 at a SNP *p*-value threshold of 1 × 10^−5^, RR 0.90 [95% CI 0.83, 0.99], *p* = 0.03 at SNP *p*-value thresholds of 1 × 10^−6^ and 1 × 10^−7^). We also observed evidence for the associations between both the mother’s and offspring’s genetic liability for JIA and repetitive behaviours at more stringent *p*-value thresholds (RR 1.13 [95% CI 1.02, 1.25], *p* = 0.02 at a SNP *p*-value threshold of 1 × 10^−3^, RR 1.15 [95% CI 1.04, 1.28], *p* = 0.01 at a SNP *p*-value threshold of 1 × 10^−4^). For further details on effect sizes and 95% CIs, please see Tables S7–S11.

PRS - polygenic risk scores, JIA - juvenile idiopathic arthritis, RR - relative risk, asd - autism cases, SCDC- social communication difficulties, coh - coherence issues, repb - repetitive behaviour, soc - sociability temperament, Threshold - SNP p-value threshold for PRS construction. As reported in Table 2, after performing MR analysis, we found little evidence of a causal role of genetic liability for rheumatoid arthritis on autism. *Q* statistics indicated little evidence of heterogeneity between the instruments according to Cochran’s *Q* for IVW (*p* = 0.16, *Q* = 47.8, df 39) and Rücker’s *Q* for MR Egger (*p* = 0.13, *Q* = 47.8, df 38). The MR-Egger intercept indicated little evidence of horizontal pleiotropy (*p* = 0.99). The mean *F* statistic was 114.08, reflecting overall good instrument strength. We also found little evidence of a causal effect of genetic liability for JIA on autism. *Q* statistics indicated little evidence of heterogeneity between the instruments according to Cochran’s *Q* for IVW (*p* = 0.63, *Q* = 3.45, df 5) and Rücker’s *Q* for MR Egger (*p* = 0.55, *Q* = 3.04, df 4). The MR-Egger intercept indicated little evidence of horizontal pleiotropy (*p* = 0.56). The mean *F* statistic was 88.43.

**Table 2:** MR analysis results assessing causal effects of rheumatoid arthritis on autism, and juvenile idiopathic arthritis on autism.

Exposure and outcome	Method	No. of SNPs	OR (95% CI)	*p*-value
*Rheumatoid arthritis and autism*				
	IVW	40	1.01 (0.98, 1.04)	0.56
	MR-Egger	40	1.01 (0.97, 1.05)	0.72
	Weighted median	40	1.00 (0.96, 1.04)	0.92
*Juvenile idiopathic arthritis and autism*				
	IVW	6	1.00 (0.98, 1.02)	0.99
	MR-Egger	6	1.02 (0.96, 1.08)	0.58
	Weighted median	6	1.00 (0.98, 1.03)	0.56

*SNPs* single nucleotide polymorphisms, *OR* odds ratio, (*95% CIs*) 95% confidence intervals, *IVW* inverse variance weighted method, *MR-Egger* Mendelian randomization Egger method.

Sensitivity analysis did not show evidence of genetic liability for autism being associated with participation during follow-up data collection in ALSPAC. For further details, please see Tables S12–15.

## Discussion

To our knowledge, this is the first large, population-based cohort study that has incorporated genomic data with phenotypic data to investigate the relationship between genetic liability to rheumatoid arthritis, autism, and autistic traits. After investigating the effects of maternal and offspring’s own genetic liability for rheumatoid arthritis on autism-related phenotypes in offspring, we found little evidence for any associations. In MR analyses, we did not find any strong evidence of causal effects.

The existing body of evidence supports an association of maternal rheumatoid arthritis with autism in the offspring [9]. The differing findings could be explained by genetic liability for and diagnosed rheumatoid arthritis being different exposures. Genetic liability represents lifetime risk, which may never manifest as a diagnosis. In our study, with the current samples available, the results we yielded show that genetic liability for rheumatoid arthritis on its own does not increase risk of autism development. Environmental exposures, epigenetic mechanisms, and possible gene-environment interactions likely also contribute to increasing risk of autism and/or autistic traits in offspring. The associations in observational studies may be partially explained by unmeasured or residual confounding. It is possible that observational findings are partially explained by the effects of antirheumatic medications. Although it has been concluded that such drugs are by and large safe to use during pregnancy [31], where appropriate, their effects on neurodevelopmental outcomes have not been investigated.

In our secondary analysis, the associations between maternal genetic liability for JIA and two autistic traits were also found but there was little evidence for a causal effect of genetic liability for JIA on autism. The observed associations appearing at more stringent *p*-value thresholds could indicate that some SNPs in these PRS may exert large effects that are diluted by SNPs included in PRS constructed at more relaxed *p*-value thresholds. However, it is also possible and more likely that due to the number of analyses being performed, false-positive associations are being observed. The lack of consistency in the direction of effects provides some evidence for the presence of false positives.

This study has several strengths. We incorporated PRSs to integrate genomic and phenotypic data, which has not been done previously with regard to this study’s questions. With individual-level genetic data from a large, prospective, population-based cohort study, we could perform analyses to investigate the genetic liability of rheumatoid arthritis carried by mothers, and also their offspring, who may have inherited additional genetic liability from their fathers. The ALSPAC study also has a rich repository of phenotypic data, including on autistic traits. Compared with non-genetic observational studies, inclusion of PRS as exposures results in less confounding as an individual’s genetic material should not be associated with sociodemographic characteristics.

We were also able to assess causality using publicly available summary-level statistics from independent GWAS in a two-sample MR analysis with larger sample sizes, resulting in increased statistical power to perform analyses for rare conditions such as autism and rheumatoid arthritis. Using data from other sample populations also enabled us to triangulate the evidence when investigating the effects of genetic liability for rheumatoid arthritis and JIA on autism-related outcomes. However, some limitations of GWAS summary statistics use are that they are often available for populations of European ancestry only and confounding due to population stratification could be present if the GWAS sample itself is not representative of its target population.

This study has other limitations. At present, only common genetic variants are identified in GWASs and thus risk of autism associated with rare genetic variants and de novo variants was not included. On their own, however, known rare and de novo variants associated with autism account for a small proportion of heritability [2]. Autism-related risk alleles may reside in sex chromosomes; this is not easily investigated with PRSs as linkage-disequilibrium structures are more complex. For the same reason, we also did not include SNPs from the MHC region, where the human leucocyte antigen HLA-DRB1 has been associated with rheumatoid arthritis (*N* = 793) and autism (*N* = 31 families) in separate genetic studies [32, 33]. For genetic studies, very large sample sizes are required to detect SNPs associated with a phenotype of interest in GWAS. JIA is a rare condition [34], as are rheumatoid arthritis and autism. With even larger GWAS sample sizes to detect a greater number of phenotype-associated SNPs, use of PRSs in epidemiology will become more powerful for assessing genetic liability of polygenic disorders and downstream health outcomes. The identification of more rheumatoid arthritis and JIA-associated SNPs in larger, future GWASs raises the possibility of associations between genetic liability for these conditions and autism.

The ALSPAC study, like other cohort studies, may have recruited participants who are on average healthier than those in the general population, and also experience attrition [35]. Both these factors may introduce selection bias. Our results from descriptive analysis indicate differences between child participants who were and were not genotyped. As shown in the sensitivity analysis, genetic liability to autism was not associated with reduced participation over time, showing consistency with previous findings [30]. The reported prevalence of autism cases included in PRS analysis is in line with numerous prevalence estimates [1]. Like many GWAS, the genotyped ALSPAC cohort is relatively homogeneous with respect to ethnicity, to avoid confounding by population stratification. We also took steps to further limit the influence of population stratification by adjusting for genetic principal components. As such, a limitation is that our findings cannot be extrapolated to individuals from other genetic ancestries. We were not able to assess maternal rheumatoid arthritis and autism-related phenotypes using observational data as there are only data available on self-reported arthritis that does not distinguish between rheumatoid and osteoarthritis.

Although we were also able to assess the genetic contribution of rheumatoid arthritis and JIA for risk of autism development, we were not able to interrogate the foetal programming hypothesis as we did not have any valid or reliable measures of inflammation from pregnancy. Sex-specific GWAS for autism to derive sex-specific PRSs for autism would also be informative in the future. No existing studies have examined the effect of rheumatoid arthritis during pregnancy directly, when the influence and levels of inflammatory activity likely differ. This could be investigated in a cohort with inflammatory biomarkers as potential mediators. Investigations need not be limited to rheumatoid arthritis if other maternal autoimmune disorders are of interest. Future studies could also explore the effects of rheumatoid arthritis medication, gene-environment interactions, or epigenetic mechanisms related to inflammatory activity or immune dysregulation that may play in a role in autism development.

## Supplementary information

Supplementary materials

## References

[1] Lai M-C, Lombardo MV, Baron-Cohen S (2014). Autism. Lancet.

[2] Lyall K, Croen L, Daniels J, Fallin MD, Ladd-Acosta C, Lee BK (2017). The changing epidemiology of autism spectrum disorders. Annu Rev Public Health.

[3] Tick B, Bolton P, Happé F, Rutter M, Rijsdijk F (2016). Heritability of autism spectrum disorders: a meta-analysis of twin studies. J Child Psychol Psychiatry.

[4] Gaugler T, Klei L, Sanders SJ, Bodea CA, Goldberg AP, Lee AB (2014). Most genetic risk for autism resides with common variation. Nat Genet.

[5] Smolen JS, Aletaha D, McInnes IB (2016). Rheumatoid arthritis. Lancet.

[6] Chen S-W, Zhong XS, Jiang LN, Zheng XY, Xiong YQ, Ma SJ (2016). Maternal autoimmune diseases and the risk of autism spectrum disorders in offspring: a systematic review and meta-analysis. Behavioural Brain Res.

[7] Wojcik S, Bernatsky S, Platt RW, Pineau CA, Clarke AE, Fombonne É (2017). Risk of autism spectrum disorders in children born to mothers with rheumatoid arthritis: a systematic literature review. Arthritis Care Res.

[8] Comi AM, Zimmerman AW, Frye VH, Law PA, Peeden JN (1999). Familial clustering of autoimmune disorders and evaluation of medical risk factors in autism. J Child Neurol.

[9] Rom AL, Wu CS, Olsen J, Jawaheer D, Hetland ML, Mørch LS (2018). Parental rheumatoid arthritis and autism spectrum disorders in offspring: a Danish nationwide cohort study. J Am Acad Child Adolesc Psychiatry.

[10] Atladóttir HO, Pedersen MG, Thorsen P, Mortensen PB, Deleuran B, Eaton WW (2009). Association of family history of autoimmune diseases and autism spectrum disorders. Pediatrics.

[11] Zhu Z, Tang S, Deng X, Wang Y (2020). Maternal systemic lupus erythematosus, rheumatoid arthritis, and risk for autism spectrum disorders in offspring: a meta-analysis. J Autism Dev Disord.

[12] Brimberg L, Sadiq A, Gregersen PK, Diamond B (2013). Brain-reactive IgG correlates with autoimmunity in mothers of a child with an autism spectrum disorder. Mol Psychiatry.

[13] Choy E (2012). Understanding the dynamics: pathways involved in the pathogenesis of rheumatoid arthritis. Rheumatology.

[14] Osokine I, Erlebacher A (2017). Inflammation and Autism: from maternal gut to fetal brain. Trends Mol Med.

[15] Enstrom AM, Van de Water JA, Ashwood P (2009). Autoimmunity in autism. Curr Opin Investig Drugs.

[16] Keil A, Daniels JL, Forssen U, Hultman C, Cnattingius S, S”derberg KC (2010). Parental autoimmune diseases associated with autism spectrum disorders in offspring. Epidemiology.

[17] Okada Y, Wu D, Trynka G, Raj T, Terao C, Ikari K (2014). Genetics of rheumatoid arthritis contributes to biology and drug discovery. Nature.

[18] Grove J, Ripke S, Als TD, Mattheisen M, Walters RK, Won H (2019). Identification of common genetic risk variants for autism spectrum disorder. Nat Genet.

[19] Murray GK, Lin T, Austin J, McGrath JJ, Hickie IB, Wray NR (2020). Could polygenic risk scores be useful in psychiatry?: A review. JAMA Psychiatry.

[20] Lawlor DA, Harbord RM, Sterne JAC, Timpson N, Davey, Smith G (2008). Mendelian randomization: using genes as instruments for making causal inferences in epidemiology. Stat Med.

[21] Boyd A, Golding J, Macleod J, Lawlor DA, Fraser A, Henderson J (2013). Cohort Profile: the ‘children of the 90s’-the index offspring of the Avon longitudinal study of parents and children. Int J Epidemiol.

[22] Fraser A, Macdonald-Wallis C, Tilling K, Boyd A, Golding J, Davey Smith G (2013). Cohort profile: the Avon longitudinal study of parents and children: ALSPAC mothers cohort. Int J Epidemiol.

[23] Williams E, Thomas K, Sidebotham H, Emond A (2008). Prevalence and characteristics of autistic spectrum disorders in the ALSPAC cohort. Developmental Med Child Neurol.

[24] Golding J, Ellis G, Gregory S, Birmingham K, Iles-Caven Y, Rai D (2017). Grand-maternal smoking in pregnancy and grandchild’s autistic traits and diagnosed autism. Sci Rep.

[25] Guyatt AL, Heron J, Knight Ble C, Golding J, Rai D (2015). Digit ratio and autism spectrum disorders in the Avon longitudinal study of parents and children: a birth cohort study. BMJ Open.

[26] Steer CD, Golding J, Bolton PF (2010). Traits contributing to the autistic spectrum. PLoS ONE.

[27] Euesden J, Lewis CM, O’Reilly PF (2015). PRSice: polygenic risk score software. Bioinformatics.

[28] Hinks A, Cobb J, Marion MC, Prahalad S, Sudman M, Bowes J (2013). Dense genotyping of immune-related disease regions identifies 14 new susceptibility loci for juvenile idiopathic arthritis. Nat Genet.

[29] *Stata Statistical Software: Release 16* [computer program]. College Station, TX: StataCorp LLC; 2019.

[30] Taylor AE, Jones HJ, Sallis H, Euesden J, Stergiakouli E, Davies NM (2018). Exploring the association of genetic factors with participation in the Avon Longitudinal Study of Parents and Children. Int J Epidemiol.

[31] Østensen M, Förger F (2013). How safe are anti-rheumatic drugs during pregnancy?. Curr Opin Pharmacol.

[32] Johnson WG, Buyske S, Mars AE, Sreenath M, Stenroos ES, Williams TA (2009). HLA-DR4 as a risk allele for autism acting in mothers of probands possibly during pregnancy. Arch Pediatrics Adolesc Med.

[33] Fries JF, Wolfe F, Apple R, Erlich H, Bugawan T, Holmes T (2002). HLA-DRB1 genotype associations in 793 white patients from a rheumatoid arthritis inception cohort: frequency, severity, and treatment bias. Arthritis Rheum.

[34] Thierry S, Fautrel B, Lemelle I, Guillemin F (2014). Prevalence and incidence of juvenile idiopathic arthritis: a systematic review. Jt Bone Spine.

[35] Boyd A, Golding J, Macleod J, Lawlor DA, Fraser A, Henderson J (2012). Cohort profile: the ‘Children of the 90s’—the index offspring of the Avon Longitudinal Study of Parents and Children. Int J Epidemiol.

