# Supplementary material for: Genetic liability to rheumatoid arthritis on autism and autistic traits: polygenic risk score and Mendelian randomization analyses
Source: Transl Psychiatry. 2022 Jan 12;12:18. doi: 10.1038/s41398-021-01772-2 (PMC8755835; doi:10.1038/s41398-021-01772-2)
Supplement: Supplementary file 1 — Supplementary materials [file 41398_2021_1772_MOESM1_ESM.docx]

## Supplementary materials

### Genetic data

Genetic data were generated from 9,912 child participants using a genome-wide SNP genotyping platform (HumanHap550-Quad; Illumina). Following quality control and imputation for missing data, genetic data for 7,977 children were available. In the cohort of mothers, 10,321 provided consent for genetic testing, resulting in genome-wide assays (Illumina 660 W-quad Beadchip). Following quality control, genetic data from 7,921 mothers were available ^1^.

Information on sociodemographic characteristics of child participants who did and did not provided genetic data are presented in Table S1. There was evidence for differences between the comparison groups with higher prevalence of autism cases and socioeconomic status (SES), and on average, lower prevalence of maternal postpartum depression and maternal smoking and higher maternal age in the group with genetic data.

**Table S1: Sociodemographic characteristics of child participants who did and did not provided genetic data**

|  |  | No genetic data | | Has genetic data | |  |
| --- | --- | --- | --- | --- | --- | --- |
| Variable of interest | | **N** | **n (%)** | **N** | **n (%)** | **p-value** |
| *Child’s biological sex* | |  |  |  |  |  |
| male | | 6843 | 3471 (50.72) | 7852 | 4027 (51.29) | 0.50 |
| female | |  | 3372 (49.28) |  | 3825 (48.71) |  |
| *Autism case status* | |  |  |  |  |  |
| yes | | 6626 | 61 (0.92) | 7849 | 101 (1.29) | 0.04 |
| *Social and communication difficulties (~10% most affected)* | | | | | | |
| yes | | 2581 | 273 (10.58) | 5450 | 518 (9.50) | 0.13 |
| *Coherence issues (~10% most affected)* | | | | | | |
| yes | | 2461 | 274 (11.13) | 5568 | 554 (9.95) | 0.11 |
| *Repetitive behaviours (~10% most affected)* | | | |  |  |  |
| yes | | 2928 | 216 (7.38) | 5557 | 385 (6.93) | 0.44 |
| *Reduced sociability temperament (~10% most affected)* | | | | | | |
| yes | | 3757 | 418 (11.13) | 6176 | 716 (11.59) | 0.48 |
| *Household income in quintiles* | | | |  |  |  |
| 0. High | | 1749 | 343 (19.61) | 4442 | 1114 (25.08) | <0.001 |
| 1. Middle high | |  | 353 (20.18) |  | 1000 (22.51) |  |
| 2. Middle | |  | 352 (20.13) |  | 909 (20.46) |  |
| 3. Middle low | |  | 345 (19.73) |  | 801 (18.03) |  |
| 4. Low | | 1749 | 356 (20.35) | 4442 | 618 (13.91) |  |
| *Parent's highest social class* | | | | | | |
| 0. professional | |  | 271 (14.76) |  | 827 (17.80) | <0.001 |
| 1. managerial and technical | |  | 762 (41.50) |  | 2148 (46.24) |  |
| 2. Skilled non-manual | |  | 500 (27.23) |  | 1058 (22.78) |  |
| 3. Skilled manual, part or unskilled | | | 303 (16.50) |  | 612 (13.18) |  |
| *Mother's educational level* | | | | | | |
| < O-level | | 5160 | 1946 (37.71) | 7075 | 1730 (24.45) | <0.001 |
| O-level | |  | 1771 (34.32) |  | 2460 (34.77) |  |
| > O-level | |  | 1443 (27.97) |  | 2885 (40.78) |  |
| *Maternal smoking since the child was 18 months old* | | | | | | |
|  | None | 3460 | 2528 (73.06) | 5888 | 4710 (79.99) | <0.001 |
| <10 | |  | 274 (7.92) |  | 405 (6.88) |  |
| 10-19 | |  | 427 (12.34) |  | 527 (8.95) |  |
| 20+ | |  | 231 (6.68) |  | 246 (4.18) |  |
| Continuous variables | |  | **Mean (sd)** |  | **Mean (sd)** |  |
| *Maternal age in years* | |  |  |  |  |  |
|  | | 6266 | 27.57 (5.07) | 7505 | 29.16 (4.74) | <0.001 |
| *Maternal Edinburgh postpartum depression score* | | | | | | |
|  | | 5021 | 7.48 (5.21) | 6906 | 6.76 (4.96) | <0.001 |

**Table S2: Associations between PRSs for rheumatoid arthritis and autism diagnosis**

|  | Mothers  (N=7685) |  | Offspring (N=7849) |  |
| --- | --- | --- | --- | --- |
| P-value threshold | RR (95% CI) | P-value | RR (95% CI) | P-value |
| 0.5 | 0.97 (0.80, 1.18) | 0.756 | 1.13 (0.92, 1.39) | 0.24 |
| 0.4 | 0.96 (0.79, 1.17) | 0.706 | 1.13 (0.92, 1.39) | 0.25 |
| 0.3 | 0.97 (0.80, 1.19) | 0.782 | 1.14 (0.93, 1.41) | 0.21 |
| 0.2 | 0.94 (0.78, 1.15) | 0.559 | 1.14 (0.92, 1.41) | 0.24 |
| 0.1 | 0.94 (0.76, 1.17) | 0.575 | 1.12 (0.89, 1.40) | 0.33 |
| 0.05 | 0.89 (0.73, 1.07) | 0.214 | 1.11 (0.88, 1.39) | 0.39 |
| 0.01 | 0.87 (0.70, 1.08) | 0.21 | 1.14 (0.91, 1.43) | 0.24 |
| 1x10^-3^ | 1.05 (0.86, 1.28) | 0.638 | 1.17 (0.96, 1.42) | 0.12 |
| 1x10^-4^ | 1.02 (0.83, 1.25) | 0.855 | 1.17 (0.97, 1.42) | 0.11 |
| 1x10^-5^ | 1.04 (0.85, 1.28) | 0.688 | 1.09 (0.91, 1.31) | 0.36 |
| 1x10^-6^ | 1.09 (0.90, 1.31) | 0.39 | 1.14 (0.95, 1.35) | 0.16 |
| 1x10^-7^ | 1.07 (0.88, 1.29) | 0.5 | 1.12 (0.94, 1.34) | 0.22 |

**Table S3: Associations between PRSs for rheumatoid arthritis and social communication difficulties**

|  | Mothers  (N=5071) |  | Offspring (N=5450) |  |
| --- | --- | --- | --- | --- |
| P-value threshold | RR (95% CI) | P-value | RR (95% CI) | P-value |
| 0.5 | 1.03 (0.95, 1.12) | 0.50 | 1.01 (0.94, 1.09) | 0.768 |
| 0.4 | 1.02 (0.94, 1.11) | 0.67 | 1.01 (0.94, 1.10) | 0.74 |
| 0.3 | 1.01 (0.93, 1.10) | 0.74 | 1.02 (0.94, 1.11) | 0.581 |
| 0.2 | 1.00 (0.92, 1.09) | 0.94 | 1.01 (0.93, 1.09) | 0.817 |
| 0.1 | 1.01 (0.93, 1.10) | 0.83 | 1.03 (0.95, 1.12) | 0.423 |
| 0.05 | 0.98 (0.90, 1.07) | 0.69 | 1.02 (0.94, 1.10) | 0.671 |
| 0.01 | 0.98 (0.90, 1.06) | 0.59 | 1.02 (0.94, 1.10) | 0.641 |
| 1x10^-3^ | 0.98 (0.90, 1.06) | 0.57 | 1.01 (0.94, 1.09) | 0.771 |
| 1x10^-4^ | 0.95 (0.87, 1.03) | 0.24 | 1.00 (0.92, 1.08) | 0.969 |
| 1x10^-5^ | 0.93 (0.85, 1.01) | 0.10 | 0.98((0.91, 1.06) | 0.626 |
| 1x10^-6^ | 0.93 (0.86, 1.02) | 0.11 | 1.01 ((0.94, 1.10) | 0.723 |
| 1x10^-7^ | 0.94 (0.86, 1.02) | 0.16 | 1.01 ((0.93, 1.10) | 0.738 |

**Table S4: Associations between PRSs for rheumatoid arthritis and coherence**

|  | Mothers  (N=4992) |  | Offspring (N=5568) |  |
| --- | --- | --- | --- | --- |
| P-value threshold | RR (95% CI) | P-value | RR (95% CI) | P-value |
| 0.5 | 1.00 (0.92, 1.09) | 0.92 | 1.05 (0.97, 1.13) | 0.26 |
| 0.4 | 1.00 (0.92, 1.09) | 0.93 | 1.05 (0.97, 1.14) | 0.23 |
| 0.3 | 1.01 (0.93, 1.10) | 0.78 | 1.06 (0.98, 1.15) | 0.13 |
| 0.2 | 1.01 (0.93, 1.09) | 0.86 | 1.06 (0.98, 1.15) | 0.16 |
| 0.1 | 1.00 (0.92, 1.09) | 0.95 | 1.07 (0.99, 1.16) | 0.11 |
| 0.05 | 0.95 (0.87, 1.03) | 0.21 | 1.01 (0.94, 1.09) | 0.76 |
| 0.01 | 0.97 (0.89, 1.06) | 0.51 | 1.05 (0.97, 1.13) | 0.24 |
| 1x10^-3^ | 1.01 (0.93, 1.10) | 0.81 | 0.99 (0.92, 1.07) | 0.78 |
| 1x10^-4^ | 1.02 ((0.93, 1.11) | 0.70 | 1.01 (0.93, 1.09) | 0.88 |
| 1x10^-5^ | 1.05 ((0.96, 1.14) | 0.29 | 1.03 (0.95, 1.11) | 0.48 |
| 1x10^-6^ | 1.06 (0.97, 1.15) | 0.20 | 1.04 (0.96, 1.12) | 0.36 |
| 1x10^-7^ | 1.06 (0.98, 1.15) | 0.17 | 1.06 (0.98, 1.15) | 0.12 |

**Table S5: Associations between PRSs for rheumatoid arthritis and repetitive behaviours**

|  | Mothers  (N=5557) |  | Offspring (N=5249) |  |
| --- | --- | --- | --- | --- |
| P-value threshold | RR (95% CI) | P-value | RR (95% CI) | P-value |
| 0.5 | 1.05 (0.95, 1.15) | 0.33 | 1.03 (0.93, 1.14) | 0.59 |
| 0.4 | 1.05 (0.95, 1.15) | 0.34 | 1.02 (0.92, 1.13) | 0.68 |
| 0.3 | 1.05 (0.96, 1.16) | 0.31 | 1.02 (0.92, 1.13) | 0.66 |
| 0.2 | 1.07 (0.97, 1.18) | 0.15 | 1.04 (0.94, 1.15) | 0.44 |
| 0.1 | 1.08 (0.98, 1.19) | 0.14 | 0.98 (0.89, 1.09) | 0.75 |
| 0.05 | 1.06 (0.97, 1.17) | 0.21 | 0.96 (0.86, 1.06) | 0.40 |
| 0.01 | 1.05 (0.96, 1.16) | 0.28 | 0.97 (0.87, 1.07) | 0.49 |
| 1x10^-3^ | 1.00 (0.91, 1.09) | 0.92 | 1.01 (0.92, 1.12) | 0.82 |
| 1x10^-4^ | 0.98 (0.89, 1.09) | 0.75 | 0.95 (0.86, 1.05) | 0.33 |
| 1x10^-5^ | 0.97 (0.88, 1.07) | 0.53 | 0.95 (0.85, 1.06) | 0.34 |
| 1x10^-6^ | 0.97 (0.88, 1.07) | 0.54 | 0.96 (0.86, 1.07) | 0.46 |
| 1x10^-7^ | 0.99 (0.90, 1.09) | 0.86 | 0.99 (0.89, 1.10) | 0.79 |

**Table S6: Associations between PRSs for rheumatoid arthritis and reduced sociability**

|  | Mothers  (N=5965) |  | Offspring (N=6176) |  |
| --- | --- | --- | --- | --- |
| P-value threshold | RR (95% CI) | P-value | RR (95% CI) | P-value |
| 0.5 | 1.00 (0.93, 1.07) | 0.96 | 0.98 (0.91, 1.05) | 0.51 |
| 0.4 | 0.99 (0.92, 1.06) | 0.80 | 0.98 (0.91, 1.05) | 0.50 |
| 0.3 | 0.99 (0.92, 1.06) | 0.71 | 0.97 (0.91, 1.04) | 0.39 |
| 0.2 | 0.98 (0.91, 1.05) | 0.56 | 0.96 (0.90, 1.03) | 0.28 |
| 0.1 | 0.97 (0.90, 1.04) | 0.39 | 0.97 (0.90, 1.04) | 0.36 |
| 0.05 | 0.96 (0.89, 1.03) | 0.27 | 0.99 (0.92, 1.06) | 0.75 |
| 0.01 | 0.96 (0.89, 1.03) | 0.21 | 1.00 (0.93, 1.07) | 0.99 |
| 1x10^-3^ | 0.99 (0.93, 1.06) | 0.82 | 1.00 (0.93, 1.07) | 0.99 |
| 1x10^-4^ | 1.04 (0.97, 1.12) | 0.26 | 1.01 (0.94, 1.08) | 0.79 |
| 1x10^-5^ | 1.05 (0.97, 1.12) | 0.21 | 1.00 (0.94, 1.07) | 0.90 |
| 1x10^-6^ | 1.02 (0.95, 1.10) | 0.53 | 1.00 (0.94, 1.07) | 0.91 |
| 1x10^-7^ | 0.99 (0.92, 1.07) | 0.87 | 1.00 (0.93, 1.07) | 0.90 |

**Table S7: Associations between PRSs for JIA and autism diagnosis**

|  | Mothers  (N=7685) |  | Offspring (N=7849) |  |
| --- | --- | --- | --- | --- |
| P-value threshold | RR (95% CI) | P-value | RR (95% CI) | P-value |
| 0.5 | 1.08 (0.89, 1.31) | 0.42 | 1.15 (0.96, 1.37) | 0.13 |
| 0.4 | 1.08 (0.89, 1.30) | 0.44 | 1.16 (0.98, 1.39) | 0.09 |
| 0.3 | 1.06 (0.87, 1.28) | 0.58 | 1.13 (0.94, 1.35) | 0.19 |
| 0.2 | 0.99 (0.82, 1.21) | 0.94 | 1.13 (0.94, 1.37) | 0.19 |
| 0.1 | 0.95 (0.79, 1.15) | 0.62 | 1.06 (0.87, 1.29) | 0.56 |
| 0.05 | 0.97 (0.80, 1.18) | 0.79 | 1.05 (0.87, 1.26) | 0.62 |
| 0.01 | 0.97 (0.79, 1.19) | 0.74 | 0.99 (0.83, 1.19) | 0.93 |
| 1x10^-3^ | 1.11 (0.90, 1.37) | 0.31 | 0.98 (0.81, 1.19) | 0.85 |
| 1x10^-4^ | 1.08 (0.88, 1.32) | 0.48 | 1.03 (0.86, 1.22) | 0.77 |
| 1x10^-5^ | 1.07 (0.90, 1.27) | 0.42 | 1.14 (0.97, 1.33) | 0.10 |
| 1x10^-6^ | 1.04 (0.87, 1.25) | 0.67 | 0.99 (0.84, 1.18) | 0.94 |
| 1x10^-7^ | 1.04 (0.87, 1.25) | 0.67 | 0.99 (0.84, 1.18) | 0.94 |

**Table S8: Associations between PRSs for JIA and social communication difficulties**

|  | Mothers  (N=5071) |  | Offspring (N=5450) |  |
| --- | --- | --- | --- | --- |
| P-value threshold | RR (95% CI) | P-value | RR (95% CI) | P-value |
| 0.5 | 1.07 (0.98, 1.16) | 0.12 | 0.99 (0.92, 1.08) | 0.90 |
| 0.4 | 1.06 (0.97, 1.15) | 0.17 | 0.99 (0.92, 1.08) | 0.90 |
| 0.3 | 1.05 (0.97, 1.14) | 0.22 | 1.00 (0.92, 1.08) | 0.94 |
| 0.2 | 1.06 (0.98, 1.15) | 0.16 | 1.02 (0.94, 1.10) | 0.71 |
| 0.1 | 1.01 (0.93, 1.10) | 0.73 | 1.01 (0.93, 1.09) | 0.88 |
| 0.05 | 1.05 (0.96, 1.13) | 0.29 | 1.00 (0.92, 1.08) | 0.95 |
| 0.01 | 1.03 (0.95, 1.12) | 0.47 | 0.98 (0.91, 1.06) | 0.61 |
| 1x10^-3^ | 0.99 (0.91, 1.08) | 0.83 | 0.98 (0.90, 1.06) | 0.61 |
| 1x10^-4^ | 0.95 (0.87, 1.03) | 0.18 | 1.00 (0.92, 1.08) | 1.00 |
| 1x10^-5^ | 0.91 (0.83, 0.99) | 0.03 | 1.00 (0.92, 1.07) | 0.82 |
| 1x10^-6^ | 0.90 (0.83, 0.99) | 0.03 | 0.96 (0.88, 1.04) | 0.27 |
| 1x10^-7^ | 0.90 (0.83, 0.99) | 0.03 | 0.96 (0.88, 1.04) | 0.27 |

**Table S9: Associations between PRSs for JIA and coherence**

|  | Mothers  (N=4992) | | Offspring  (N=5568) | |
| --- | --- | --- | --- | --- |
| P-value threshold | RR (95% CI) | P-value | RR (95% CI) | P-value |
| 0.5 | 1.04 (0.96, 1.14) | 0.34 | 1.02 (0.94, 1.10) | 0.70 |
| 0.4 | 1.05 (0.96, 1.15) | 0.28 | 1.03 (0.95, 1.11) | 0.52 |
| 0.3 | 1.06 (0.97, 1.15) | 0.20 | 1.04 (0.96, 1.13) | 0.32 |
| 0.2 | 1.06 (0.97, 1.15) | 0.20 | 1.04 (0.96, 1.13) | 0.29 |
| 0.1 | 1.07 (0.98, 1.17) | 0.14 | 1.04 (0.96, 1.13) | 0.30 |
| 0.05 | 1.07 (0.98, 1.17) | 0.11 | 1.03 (0.96, 1.12) | 0.41 |
| 0.01 | 1.06 (0.97, 1.16) | 0.18 | 1.09 (1.00, 1.18) | 0.04 |
| 1x10^-3^ | 1.05 (0.97, 1.14) | 0.23 | 1.03 (0.95, 1.12) | 0.47 |
| 1x10^-4^ | 1.01 (0.93, 1.09) | 0.84 | 1.03 (0.95, 1.12) | 0.46 |
| 1x10^-5^ | 0.99 (0.91, 1.08) | 0.78 | 1.06 (0.98, 1.14) | 0.16 |
| 1x10^-6^ | 1.01 (0.93, 1.09) | 0.89 | 1.02 (0.94, 1.10) | 0.65 |
| 1x10^-7^ | 1.01 (0.93, 1.09) | 0.89 | 1.02 (0.94, 1.10) | 0.65 |

**Table S10: Associations between PRSs for JIA and repetitive behaviours**

|  | Mothers  (N=5249) | | Offspring  (N=5557) | |
| --- | --- | --- | --- | --- |
| P-value threshold | RR (95% CI) | P-value | RR (95% CI) | P-value |
| 0.5 | 1.00 (0.90, 1.11) | 0.98 | 1.01 (0.91, 1.12) | 0.88 |
| 0.4 | 1.00 (0.90, 1.11) | 0.94 | 1.00 (0.90, 1.10) | 0.93 |
| 0.3 | 0.97 (0.87, 1.07) | 0.53 | 1.00 (0.90, 1.10) | 0.97 |
| 0.2 | 0.97 (0.87, 1.07) | 0.53 | 1.01 (0.92, 1.12) | 0.78 |
| 0.1 | 0.97 (0.88, 1.08) | 0.62 | 1.03 (0.93, 1.15) | 0.51 |
| 0.05 | 1.01 (0.91, 1.12) | 0.84 | 1.07 (0.97, 1.18) | 0.20 |
| 0.01 | 1.03 (0.93, 1.15) | 0.55 | 1.06 (0.96, 1.16) | 0.28 |
| 1x10^-3^ | 1.13 (1.02, 1.25) | 0.02 | 1.07 (0.97, 1.18) | 0.19 |
| 1x10^-4^ | 1.15 (1.04, 1.28) | 0.01 | 1.13 (1.02, 1.24) | 0.02 |
| 1x10^-5^ | 1.07 (0.96, 1.18) | 0.21 | 1.08 (0.97, 1.19) | 0.15 |
| 1x10^-6^ | 1.06 (0.96, 1.18) | 0.25 | 1.07 (0.97, 1.17) | 0.18 |
| 1x10^-7^ | 1.06 (0.96, 1.18) | 0.25 | 1.07 (0.97, 1.17) | 0.18 |

**Table S11: Associations between PRSs for JIA and reduced sociability**

|  | Mothers  (N=5249) | | Offspring  (N=5557) | |
| --- | --- | --- | --- | --- |
| P-value threshold | RR (95% CI) | P-value | RR (95% CI) | P-value |
| 0.5 | 1.00 (0.90, 1.11) | 0.98 | 1.01 (0.91, 1.12) | 0.88 |
| 0.4 | 1.00 (0.90, 1.11) | 0.94 | 1.00 (0.90, 1.10) | 0.93 |
| 0.3 | 0.97 (0.87, 1.07) | 0.53 | 1.00 (0.90, 1.10) | 0.97 |
| 0.2 | 0.97 (0.87, 1.07) | 0.53 | 1.01 (0.92, 1.12) | 0.78 |
| 0.1 | 0.97 (0.88, 1.08) | 0.62 | 1.03 (0.93, 1.15) | 0.51 |
| 0.05 | 1.01 (0.91, 1.12) | 0.84 | 1.07 (0.97, 1.18) | 0.20 |
| 0.01 | 1.03 (0.93, 1.15) | 0.55 | 1.06 (0.96, 1.16) | 0.28 |
| 1x10^-3^ | 1.13 (1.02, 1.25) | 0.02 | 1.07 (0.97, 1.18) | 0.19 |
| 1x10^-4^ | 1.15 (1.04, 1.28) | 0.01 | 1.13 (1.02, 1.24) | 0.02 |
| 1x10^-5^ | 1.07 (0.96, 1.18) | 0.21 | 1.08 (0.97, 1.19) | 0.15 |
| 1x10^-6^ | 1.06 (0.96, 1.18) | 0.25 | 1.07 (0.97, 1.17) | 0.18 |
| 1x10^-7^ | 1.06 (0.96, 1.18) | 0.25 | 1.07 (0.97, 1.17) | 0.18 |

**Table S12: Associations between PRSs for autism (mothers) and questionnaire participation. Unadjusted and adjusted for 10 genetic principal components (N=7478)**

|  | Unadjusted | | Adjusted | |
| --- | --- | --- | --- | --- |
| P-value threshold | β (95%CI) | P-value | β (95%CI) | P-value |
| 0.5 | 0.05 (-0.07, 0.16) | 0.42 | 0.05 (-0.07, 0.16) | 0.40 |
| 0.4 | 0.04 (-0.08, 0.15) | 0.51 | 0.04 (-0.08, 0.16) | 0.49 |
| 0.3 | 0.05 (-0.07, 0.16) | 0.43 | 0.05 (-0.07, 0.16) | 0.41 |
| 0.2 | 0.07 (-0.05 0.19) | 0.24 | 0.07 (-0.04, 0.19) | 0.22 |
| 0.1 | 0.07 (-0.04, 0.19) | 0.22 | 0.08 (-0.04, 0.19) | 0.20 |
| 0.05 | 0.06 (-0.06, 0.17) | 0.35 | 0.06 (-0.06, 0.18) | 0.30 |
| 0.01 | 0.05 (-0.06, 0.17) | 0.38 | 0.05 (-0.06, 0.17) | 0.37 |
| 1x10^-3^ | 0.06 (-0.06, 0.18) | 0.30 | 0.05 (-0.06, 0.17) | 0.36 |
| 1x10^-4^ | 0.01 (-0.10, 0.13) | 0.81 | 0.01 (-0.11, 0.13) | 0.93 |
| 1x10^-5^ | -0.04 (-0.16, 0.07) | 0.49 | -0.05 (-0.16, 0.07) | 0.45 |
| 1x10^-6^ | -0.05 ( -0.17, 0.06) | 0.40 | -0.05 (-0.17, 0.07) | 0.40 |
| 1x10^-7^ | 0.06 (-0.06, 0.18) | 0.30 | 0.06 (-0.05, 0.18) | 0.28 |

**Table S13: Associations between PRSs for autism (mothers) and total participation in questionnaires and clinic. Unadjusted and adjusted for 10 genetic principal components (N=7478)**

|  | Unadjusted | | Adjusted | |
| --- | --- | --- | --- | --- |
| P-value threshold | β (95%CI) | P-value | β (95%CI) | P-value |
| 0.5 | 0.05 (-0.07, 0.17) | 0.44 | 0.05 (-0.07, 0.17) | 0.43 |
| 0.4 | 0.04 (-0.08, 0.16) | 0.54 | 0.04 (-0.08, 0.16) | 0.52 |
| 0.3 | 0.05 (-0.08, 0.17) | 0.45 | 0.05 (-0.07, 0.17) | 0.42 |
| 0.2 | 0.07 (-0.05, 0.19) | 0.25 | 0.07 (-0.05, 0.20) | 0.23 |
| 0.1 | 0.07 (-0.05, 0.20) | 0.23 | 0.08 (-0.04, 0.20) | 0.21 |
| 0.05 | 0.06 (-0.07, 0.18) | 0.37 | 0.06 (-0.06, 0.18) | 0.32 |
| 0.01 | 0.05 (-0.07, 0.17) | 0.41 | 0.05 (-0.07, 0.18) | 0.40 |
| 1x10^-3^ | 0.06 (-0.07, 0.18) | 0.38 | 0.05 (-0.07, 0.17) | 0.44 |
| 1x10^-4^ | 0.01 (-0.12, 0.13) | 0.89 | -0.01 (-0.13, 0.12) | 0.90 |
| 1x10^-5^ | -0.04 (-0.17, 0.08) | 0.50 | -0.05 (-0.17, 0.08) | 0.46 |
| 1x10^-6^ | -0.05 (-0.17, 0.07) | 0.43 | -0.05 (-0.17, 0.07) | 0.42 |
| 1x10^-7^ | 0.07(-0.06, 0.19) | 0.29 | 0.07 (-0.06, 0.19) | 0.28 |

**Table S14: Associations between PRSs for autism (child) and questionnaire participation (child-based and child completed).** **Unadjusted and adjusted for 10 genetic principal components + sex (N=7,499)**

|  | Unadjusted | | Adjusted | |
| --- | --- | --- | --- | --- |
| P-value threshold | β (95%CI) | P-value | β (95%CI) | P-value |
| 0.5 | -0.04 (-0.37, 0.29) | 0.82 | -0.02 (-0.35, 0.31) | 0.90 |
| 0.4 | -0.04 (-0.37, 0.30) | 0.83 | -0.02 (-0.34, 0.31) | 0.92 |
| 0.3 | -0.04 (-0.37, 0.29) | 0.81 | -0.03 (-0.36, 0.29) | 0.84 |
| 0.2 | -0.03 (-0.36, 0.30) | 0.87 | -0.01 (-0.34, 0.32) | 0.95 |
| 0.1 | -0.07 (-0.40, 0.26) | 0.67 | -0.04 (-0.37, 0.28) | 0.79 |
| 0.05 | -0.07 (-0.40, 0.26) | 0.68 | -0.06 (-0.39, 0.27) | 0.72 |
| 0.01 | -0.01 (-0.33, 0.33) | 0.99 | 0.004 (-0.32,0.33) | 0.98 |
| 1x10^-3^ | 0.25 (-0.08, 0.57) | 0.14 | 0.30 (-0.03, 0.62) | 0.07 |
| 1x10^-4^ | -0.23 (-0.56, 0.10) | 0.17 | -0.19 (-0.52, 0.13) | 0.25 |
| 1x10^-5^ | -0.13 (-0.46, 0.20) | 0.45 | -0.09 (-0.42, 0.23) | 0.57 |
| 1x10^-6^ | -0.26 (-0.60, 0.08) | 0.13 | -0.20 (-0.54, 0.13) | 0.24 |
| 1x10^-7^ | 0.27 (-0.06, 0.60) | 0.11 | 0.28 (-0.05, 0.61) | 0.09 |

**Table S15: Associations between PRSs for autism (child) and total participation in questionnaires and clinics. Unadjusted and adjusted for 10 genetic principal components + sex (N= 7,499)**

|  | Unadjusted | | Adjusted | |
| --- | --- | --- | --- | --- |
| P-value threshold | β (95%CI) | P-value | β (95%CI) | P-value |
| 0.5 | -0.07 (-0.46, 0.31) | 0.70 | -0.05 (-0.43, 0.33) | 0.80 |
| 0.4 | -0.07 (-0.46, 0.31) | 0.70 | -0.05 (-0.43, 0.33) | 0.80 |
| 0.3 | -0.08 (-0.46, 0.30) | 0.67 | -0.07 (-0.45, 0.31) | 0.72 |
| 0.2 | -0.07 (-0.45, 0.31) | 0.71 | -0.05 (-0.43, 0.33) | 0.80 |
| 0.1 | -0.12 (-0.51, 0.26) | 0.52 | -0.09 (-0.46, 0.29) | 0.64 |
| 0.05 | -0.12 (-0.50, 0.26) | 0.54 | -0.11 (-0.48, 0.27) | 0.58 |
| 0.01 | -0.03 (-0.42, 0.35) | 0.86 | -0.02 (-0.40, 0.35) | 0.91 |
| 1x10^-3^ | 0.26 (-0.12, 0.64) | 0.18 | 0.32 (-0.05, 0.69) | 0.09 |
| 1x10^-4^ | -0.32 (-0.70, 0.06) | 0.10 | -0.26 (-0.64, 0.11) | 0.17 |
| 1x10^-5^ | -0.13 (-0.52, 0.25) | 0.49 | -0.09 (-0.47, 0.28) | 0.62 |
| 1x10^-6^ | -0.29 (-0.68, 0.10) | 0.15 | -0.22 (-0.61, 0.17) | 0.27 |
| 1x10^-7^ | 0.33 (-0.05, 0.71) | 0.09 | 0.34 (-0.04, 0.72) | 0.08 |

### MR – sensitivity checks

To test for heterogeneity between instrument effect sizes, we calculated the Cochran’s *Q* statistic for the IVW method and Rücker’s *Q* statistic for the MR Egger method. We also assessed for the presence of horizontal pleiotropy by evaluating whether the MR Egger intercept passed through 0. Rücker’s *Q* for the MR-Egger method was used to identify any residual horizontal pleiotropy^2^. All MR analyses assume no measurement error (NOME) in the SNP-exposure association estimates, or at least that their variances are negligible^3^. To quantify the degree of NOME violation in the IVW regression, we used the formula: mean *F* statistic minus 1 divided by the mean *F* statistic. The *I^2^_GX_* statistic was calculated to quantify the degree of NOME violation in the MR-Egger regression, both weighted and unweighted versions. These statistics can be quantified on a scale from 0-1, with higher values indicating less influence by measurement error. We also calculated the mean *F* statistic to assess the strength of all the IVs included in the MR analysis. All MR analyses was performed using RStudio 3.6.3 and the TwoSampleMR package^4^. To assess the causal effects of genetic liability for JIA and autism, we repeated the analyses described above.

For the analysis assessing rheumatoid arthritis on autism, there was little evidence for directional, horizontal pleiotropy (MR Egger intercept =8.5x10^-5^, p=0.99). Following tests to probe the NOME assumption, the IVW method performed well with a score of 0.99, reflecting very little regression dilution bias. The unweighted and weighted I^2^_GX_ statistics, 0.98 and 0.97 respectively, also indicating little violation of the NOME assumption.

### References

1. Fraser A, et al. Cohort Profile: the Avon Longitudinal Study of Parents and Children: ALSPAC mothers cohort. *International journal of epidemiology.* 2013;42(1):97-110.

2. Bowden J, Hemani G, Davey Smith G. Invited Commentary: Detecting Individual and Global Horizontal Pleiotropy in Mendelian Randomization—A Job for the Humble Heterogeneity Statistic? *Am J Epidemiol.* 2018;187(12):2681-2685.

3. Bowden J,et al. Assessing the suitability of summary data for two-sample Mendelian randomization analyses using MR-Egger regression: the role of the I2 statistic. *International journal of epidemiology.* 2016;45(6):1961-1974.

4. Hemani G, et al. The MR-Base platform supports systematic causal inference across the human phenome. *eLife.* 2018;7:e34408.
